# Hemolytic anemia caused by kinked graft 6 months after aortic dissection repair

**DOI:** 10.1186/s13019-022-02079-z

**Published:** 2022-12-14

**Authors:** Kazuki Tamura, Wataru Tatsuishi, Yasunobu Konishi, Naoki Konno, Yusuke Kato, Tomonobu Abe

**Affiliations:** Division of Cardiovascular Surgery, Department of General Surgical Science, Gunma University, 3-39-15 Showa, Maebashi, Gunma 371-8511 Japan

**Keywords:** Hemolytic anemia, Aortic dissection, Ascending aorta, Surgical

## Abstract

**Background:**

Clinically insignificant hemolytic anemia is occasionally a complication of prosthetic valve replacement. However, hemolysis related to kinked grafts is a very rare complication after central repair for acute aortic dissection.

**Case presentation:**

A 42-year-old man had undergone replacement of the ascending aorta and a root repair for type A aortic dissection 6 months previously. Laboratory data showed mild hemolysis 5 months later, and he began to complain of fatigue on exertion. The serum hemoglobin level reduced to 8.6 g/dL, and lactate dehydrogenase levels increased to 3071 IU/L with gross change in urine color, indicating hemoglobinuria. We diagnosed mechanical hemolytic anemia caused by a kinked graft and planned a repeat operation. The kinked graft was resected and graft-graft anastomosis was performed. Postoperatively, the clinical course was uneventful, and the hemolytic anemia completely resolved.

**Conclusion:**

We herein report a case of hemolytic anemia caused by kinking of the graft 6 months after acute aortic dissection repair. The diagnosis was swiftly made, and the patient was successfully managed with redo surgery.

## Background

Symptomatic lysis of red blood cells (RBCs) caused by a kinked graft after central repair for acute aortic dissection is rare and is primarily attributed to mechanical damage as a result of high shear stress, turbulent flow, and physical interaction. Treating hemolysis is important because once it exacerbates, the general condition may become unstable, leading to a critical situation. We report a case of severe hemolytic anemia unrelated to a valve prosthesis 6 months after aortic dissection repair.

## Case presentation

**Fig. 1:**
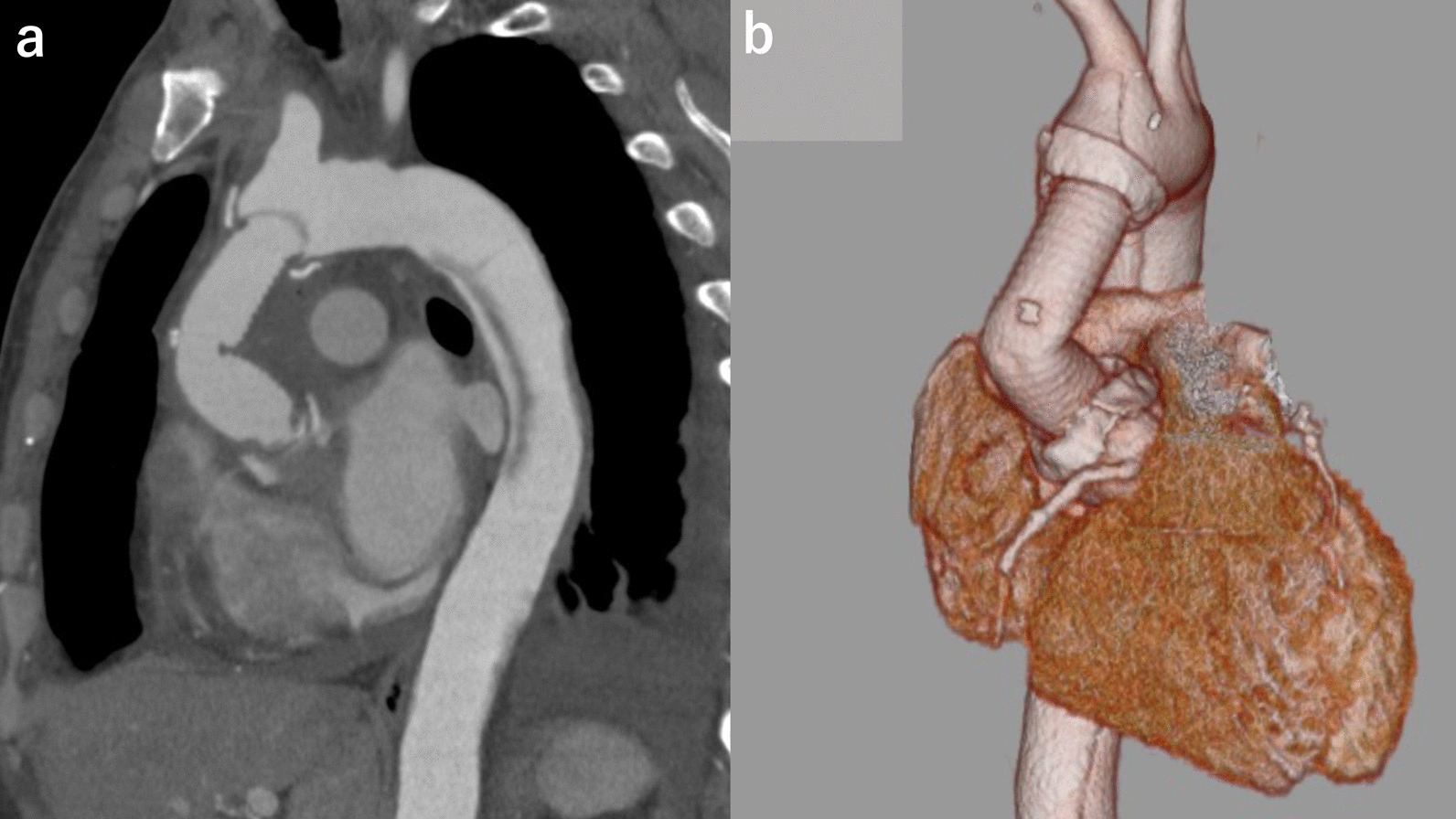
Chest CT scan after initial surgery. **a** saggital view. **b** 3D-CT

**Fig. 2:**
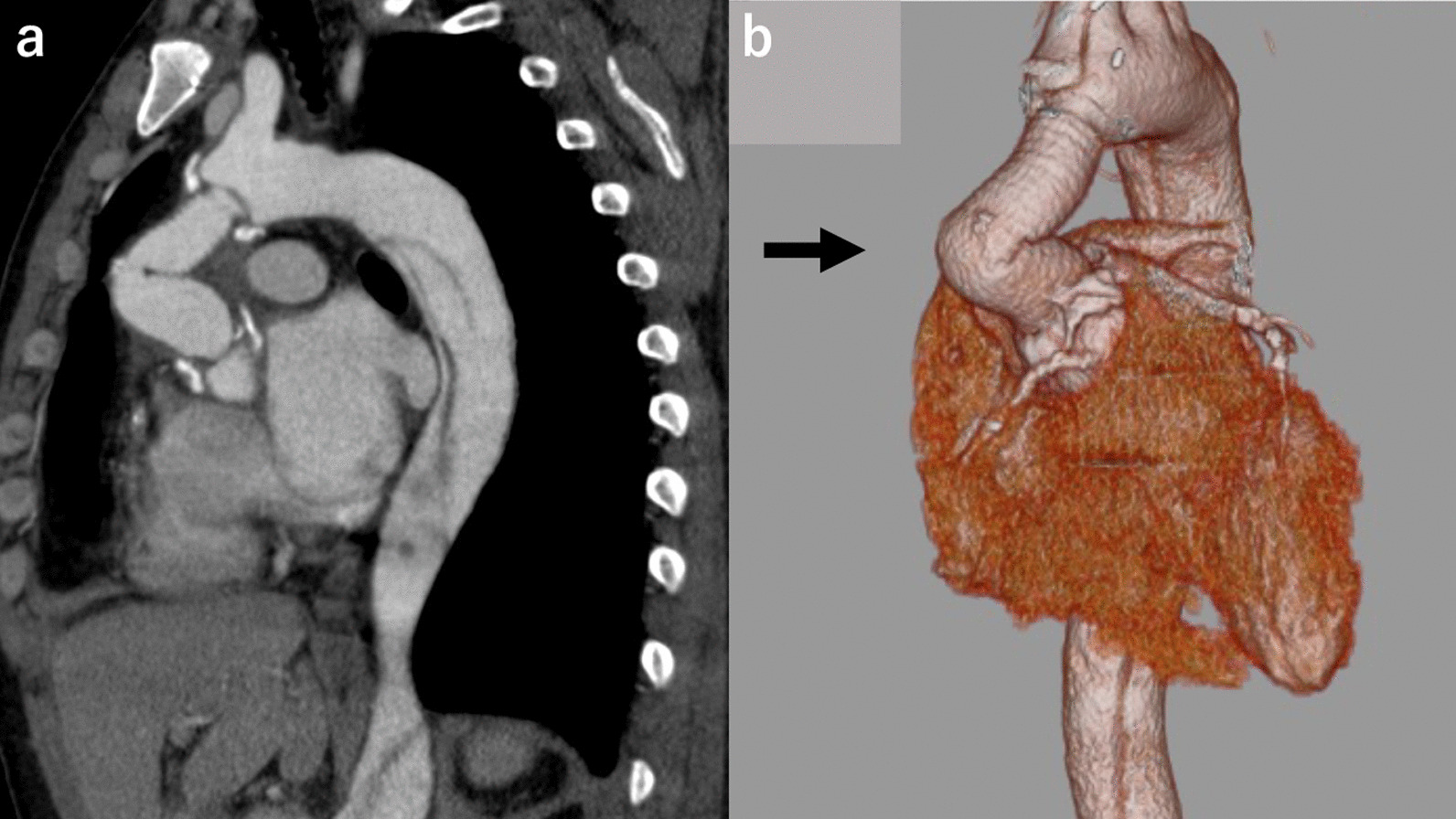
Chest CT scan before redo surgery showed severe kinking of the graft (arrow) approximately 35 mm from the proximal anastomosis. **a** saggital view. **b** 3D-CT

A 42-year-old man underwent emergency ascending aortic replacement (Triplex Advanced; Terumo Corporation, Tokyo, Japan, 22 mm and 20 mm) and aortic valve resuspension for acute type A aortic dissection at our institution 6 months previously. During the primary surgery, distal anastomosis was performed using only the outer felt, and bovine pericardium was used to cover the site with the intimal tear proximally as root intima patch repair. Replacement of the ascending aorta was performed using a 20-mm graft, and the proximal part had a 22 mm graft-graft anastomosis. The operation and postoperative course were uneventful, and the patient was discharged and followed up monthly. (Fig. 1a, b) Four months later, there was a slight increase in the serum lactate dehydrogenase (LDH) level. At 5 months after the surgery, laboratory data indicated mild hemolysis, and he complained of fatigue on exertion. Six months after the surgery, he was admitted to our institution because of severe hemolysis that required blood transfusion. The serum hemoglobin (Hb) level was 8.6 g/dL, LDH enzyme level: 3071 IU/L, hematocrit: 25.8%, relative reticulocyte count: 8.1%, total bilirubin: 4.1 mg/dL, blood urea nitrogen (BUN): 13 mg/dL, and creatinine: 1.38 mg/dL. Upon discharge after the primary surgery, the serum BUN level was 14 mg/dL, and creatinine was 0.75 mg/dL. A peripheral blood smear showed many schistocytes; however, the Coombs test was negative. Physical examination showed yellowish skin discoloration and red-yellow urine color, while eye examination showed jaundice and conjunctival pallor. A systolic murmur (Levine II) was heard in the second right intercostal space. Transthoracic echocardiography (TTE) revealed a severely kinked graft in the ascending aorta, with normal left ventricular function and valve. At the kinked site, the peak jet velocity was 4.8 m/sec and the pressure gradient was 92 mmHg. Computed tomography showed severe kinking at the graft, approximately 35 mm from the proximal anastomosis (Fig. 2a, b). These data suggested that mechanical destruction of red blood cells by the kinked graft caused the hemolytic anemia, and he needed resection of the replaced ascending aorta. He required transfusion of 14 units of blood in the 4 days before the surgery, and the Hb was 8.6 g/dL at the time of surgery.

**Fig. 3:**
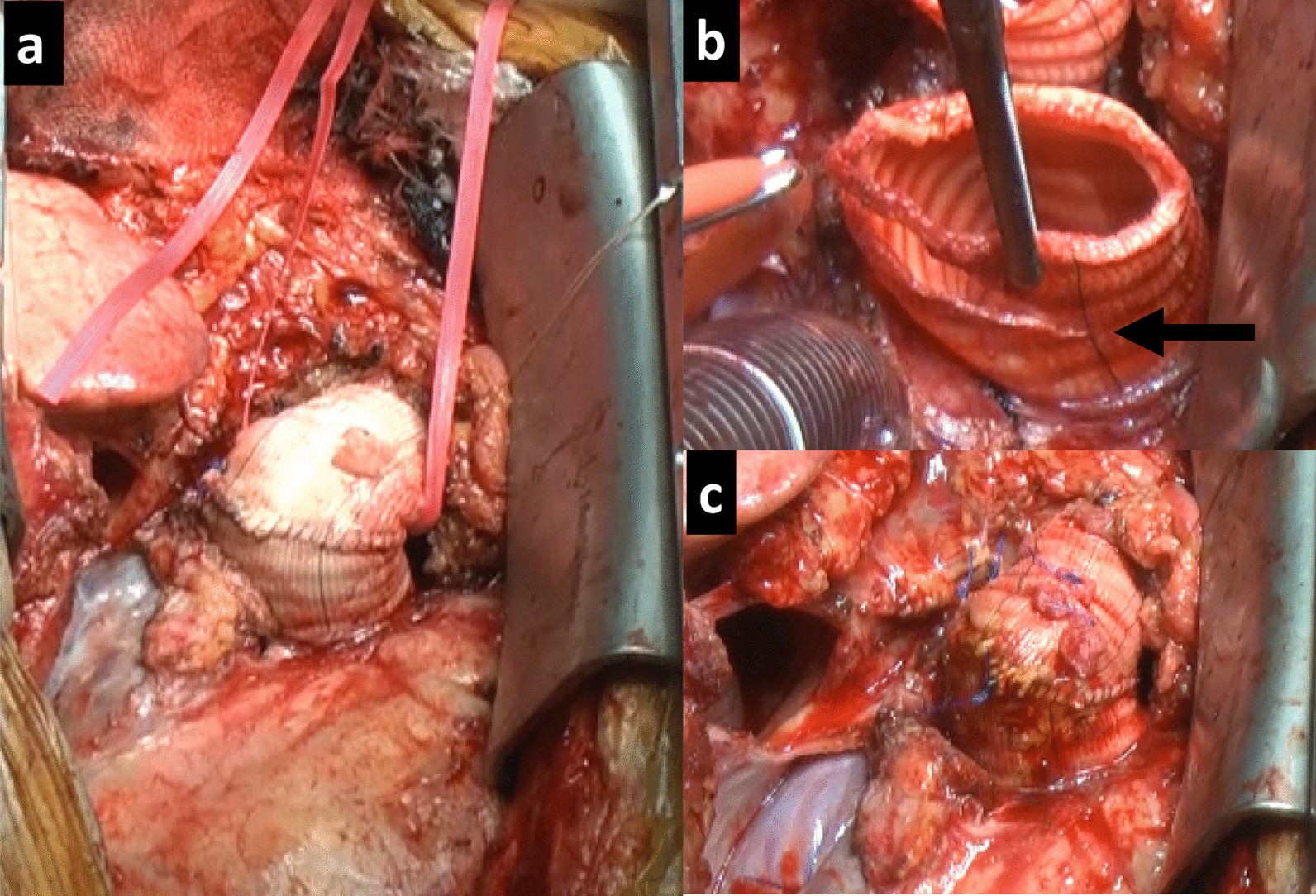
Intraoperative inspection. **a** Slightly distal to the valve level, the graft showed a sharp, crinkled oblique fold. **b** The proximal and distal sides of the kinked graft were resected diagonally (arrow). **c** Graft-graft anastomosis of approximately 1 cm in length was performed

The chest was opened by redo median sternotomy. Slightly distal to the valve level, the graft showed a sharp, crinkled oblique fold partially obstructing the post-valvular flow, and a strong thrill was felt at the site of the kinked graft (Fig. 3a). Extracorporeal circulation was established by femoral artery cannulation and venous drainage from the right atrium. After inserting the left ventricular vent cannula through the left superior pulmonary vein, clamping, and anterograde cardioplegia, the graft was opened at the suture line. The valve showed normal function with no regurgitation. The proximal and distal sides of the kinked graft were resected diagonally, and graft-graft anastomosis of approximately 1 cm in length was performed (Fig. 3b, c). The thrill became weaker than before resection after unclamping. The duration of extracorporeal circulation was 66 min, and the operation time was 283 min.

**Fig. 4:**
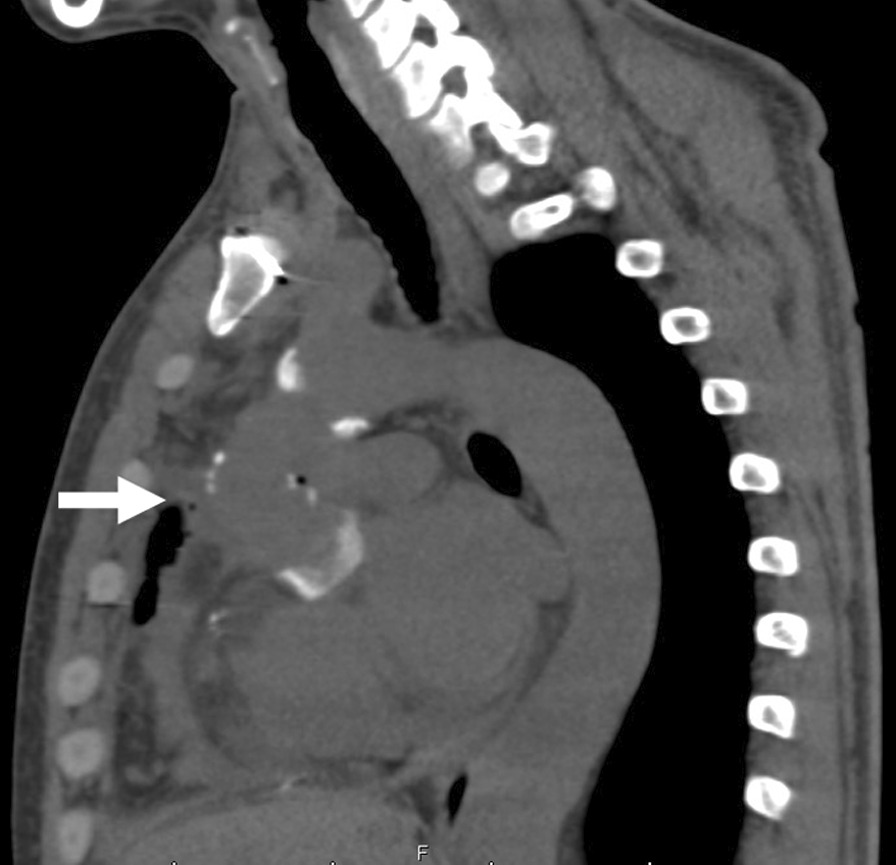
Chest CT scan 5 days after redo surgery showed no obstruction in the ascending aorta (arrow)

Postoperatively, gross hematuria and hemolytic anemia resolved completely and the serum LDH and bilirubin levels normalized (Table 1). Subsequent surgical and clinical course were uneventful, with discharge in very good condition after eight days (Fig. 4).

**Table 1:** Summary of laboratory data

	1 month after initial surgery	5 months after initial surgery	6 months after initial surgery	1 day before redo surgery	8 days after redo surgery	3 months after redo surgery
Hb (g/dL)	11.4	9.5	4.7	8.6	9	13.3
LDH (U/L)	249	1960	3898	3071	820	210
T-Bil (mg/dL)	0.6	2.5	4.6	3.8	1.1	0.4
BUN (mg/dL)	15	17.8	18.7	13	15	19.7
Cre (mg/dL)	0.81	1.01	1.61	1.38	1.05	0.81

Hemolytic anemia resolved completely and the serum LDH and bilirubin levels normalized. Kidney injury has resolved

*BUN*, blood urea nitrogen; *Cre*, creatinine; *Hb*, hemoglobin; *LDH*, lactate dehydrogenase; *T-Bil*, total bilirubin

## Discussion

This was a rare case of hemolytic anemia caused by kinking of a graft following surgery for aortic dissection. Hemolytic anemia after open heart surgery is known to be caused mainly by perivalvular leakage [1]. Hemolytic anemia after central repair for acute aortic dissection has been attributed to outer compression of the graft, disturbance of blood flow at the site of obstruction, and a stenotic lumen within the graft. Davison et al. [2] and Stanger et al. [3] reported that graft stenosis is mainly due to the inversion of inner felt strips (50%) and not by kinking, as in our case [2, 3]. In this case, we diagnosed hemolytic anemia due to graft kinking because no inner felt or artificial valve was used during the initial surgery.

The magnitude of turbulent shear stresses in the flow field and the physical interaction of red blood cells (RBCs) with prosthesis are major factors in hemolytic anemia induced by cardiovascular prosthetic devices [4]. The most common hemolytic threshold value for RBC damage was reported to be 400 N/m^2^ by Sallam and Hwang [5]. Lu et al. determined the hemolytic threshold value for RBC damage to be between 400 and 800 N/m^2^, and it may depend on high shear stress and exposure times to the shear fields [4]. High turbulent shear stress is present at locations with high-velocity gradients and at locations immediately distal to the valve leaflets. It could be expected that these flow turbulences might be intensified by a sharp fold just distal to the valve, thereby inducing turbulent shear stress on its own and causing severe RBC damage and clinically relevant hemolysis, as in our case [3]. In this case, it was expected that the increased shear stress due to the high degree of flexion was the cause of the hemolytic anemia, and the most effective treatment was the replacement of the kinked area by reoperation.

**Fig. 5:**
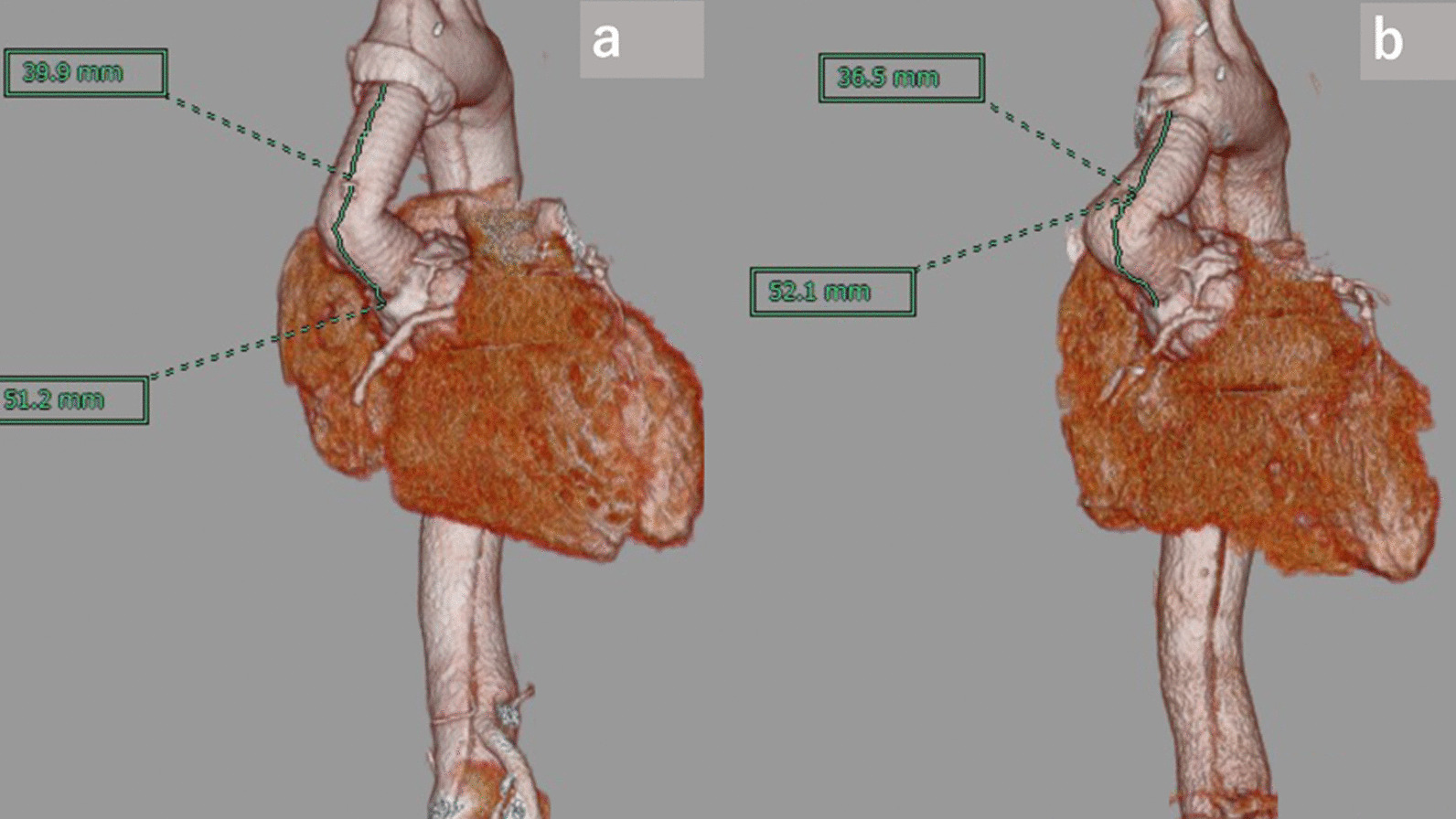
The lengths of the grafts. **a** after initial surgery. **b** after redo surgery

We speculate that kinking of the aortic graft may be related to a relatively long graft length. The computed tomography (CT) images immediately after the initial aortic dissection surgery showed mild flexion of the graft, and there were no signs of hemolytic anemia at that time. Sabzi et al. [6] determined, by applying pressure to both ventricles during surgery, the appropriate anastomotic position and optimal graft length. Takami et al. reported that the Gelseal™ graft increased by approximately 26% compared with the package size when used in ascending aorta surgery [7]. Mattens et al. reported that the diameter of the Gelseal™ graft increased by 31.4% at 2 years after implantation in the descending thoracic aorta [8]. Hori et al. reported that Triplex showed the greatest increase in diameter among Hemashield, J-Graft, and Triplex after implantation compared to the package size within 43 months of surgery. In addition to reports of cases of flexion due to elongation of the grafts [2, 9–12], Zierer et al. reported enlargement of the residual aorta after the repair of acute type A aortic dissection, and this is suggested to be one of the reasons for kinked grafts [13]. However, the increase of the aortic graft length was not observed in our case, though it was not electrocardiogram synchronized (Fig. 5a, b). Postoperative changes in the surrounding tissues such as the cardiac position and the aortic arch may have affected the kinking of the graft. In cases of hemolytic anemia after the use of a graft, it is necessary to consider kinking of the graft as a possible cause.

## Conclusion

We encountered a case of severe hemolytic anemia due to severe kinking of the graft 6 months after aortic dissection surgery. The kinking was resolved by reoperation, and the postoperative course was good. The graft length should be carefully determined during the first surgery for aortic dissection.

## Data Availability

Not applicable.

## Abbreviations

LDH: Lactate dehydrogenase
Hb: Hemoglobin
BUN: Blood urea nitrogen
TTE: Transthoracic echocardiography
CT: Computed tomography
